# An Uncommon Presentation of a Common Disease: Hoarseness of Voice in a Young Patient With Tuberculosis

**DOI:** 10.7759/cureus.89072

**Published:** 2025-07-30

**Authors:** Noor Ehsan, Maha Batool, Imran Farooka, Mahmood N Malik

**Affiliations:** 1 Internal Medicine, Gulab Devi Teaching Hospital, Lahore, PAK; 2 Internal Medicine, Gulab Devi Teaching Hospital, Al-Aleem Medical College, Lahore, PAK

**Keywords:** extrapulmonary tuberculosis, hoarseness of voice, lymph node tuberculosis, recurrent laryngeal nerve, tuberculosis, vocal cord paralysis

## Abstract

Pulmonary and extrapulmonary tuberculosis impose a major load of chronic disease burden. Lymph node and pleural tuberculosis are the most common types of extrapulmonary tuberculosis. Isolated hoarseness of voice is a rare presenting feature of lymph node tuberculosis. We present the case of a young girl who presented to us with the complaint of hoarseness of voice for two months. A contrast-enhanced CT of the neck and chest revealed bilateral cervical and mediastinal lymphadenopathy encroaching on the aortopulmonary window. A 70-degree rigid endoscopy revealed left vocal cord paralysis. A cervical lymph node excision biopsy showed necrotizing granulomatous inflammation and Langhan-type giant cells. The diagnosis was confirmed by culture on Löwenstein-Jensen medium and drug susceptibility testing, which identified *Mycobacterium tuberculosis* sensitive to all first-line antitubercular drugs. She was put on weight-based antitubercular therapy, after which her voice showed improvement, and she gained four kilograms. We present this case to highlight the importance of being familiar with uncommon presentations of common diseases, particularly in areas of high disease endemicity, to allow for timely diagnosis and treatment.

## Introduction

Tuberculosis is a chronic, communicable disease that poses a major threat to vulnerable population groups [1]. In 2023, 10.8 million new cases of tuberculosis were reported worldwide, and it claimed the lives of 1.25 million people. Pakistan is one of the five countries with the highest disease burden in the world [2]. Along with pulmonary tuberculosis, extrapulmonary tuberculosis is also an important cause of morbidity and mortality, being responsible for 15 percent of all the diagnosed cases of tuberculosis worldwide. Lymph node and pleural tuberculosis are the leading types of extrapulmonary tuberculosis [3]. Sometimes tuberculosis manifests with symptoms and signs that overlap with other illnesses, like sarcoidosis or lymphoma. Sarcoidosis and lymphoma can also present with lymphadenopathy, fever, weight loss, and compressive symptoms resulting from enlarged lymph nodes [4,5]. In such cases, the diagnosis and treatment of tuberculosis may be delayed. This case report highlights an unusual presentation of tuberculosis where the patient presented with the sole complaint of hoarseness of voice [6]. Although uncommon, hoarseness of voice can be a presenting symptom of tuberculosis. This may result from apical lung fibrosis, entrapment of the recurrent laryngeal nerve, or compression of the nerve by enlarged mediastinal lymph nodes [7,8]. This particular case of lymph node tuberculosis highlights the importance of being familiar with uncommon presentations of common diseases, like tuberculosis, particularly in areas where the disease prevalence is high. Our patient was diagnosed with tuberculous lymphadenitis based on a lymph node excision biopsy, histopathology, and culture reports, and was put on weight-based first-line anti-tubercular therapy (ATT). 

## Case presentation

A 15-year-old girl reported to the Medical Outpatient Department of Gulab Devi Teaching Hospital, Lahore, Pakistan, in October 2024. Her only complaint was hoarseness of voice, which she'd had for two months. Hoarseness of voice was gradual in onset and progressively increasing, not associated with throat pain, cough, sputum, post-nasal dripping, voice fatigue, dysphagia, shortness of breath on exertion, hemoptysis, fever, weight loss or weight gain, night sweats, increased somnolence, cold intolerance, or menstrual irregularities. She had no history of regurgitation of food, heartburn, halitosis, weakness in any part of the body, double vision, or voice overuse. She was a non-smoker and had never undergone any surgery or endotracheal intubation. She did not have a history of tuberculosis in the past, but she had a history of exposure to tuberculosis through a family member, who had gotten treated for pulmonary tuberculosis a year ago.

The patient had a thin build, conjunctival pallor, some temporal wasting, and bilaterally enlarged, matted cervical lymph nodes, which were mobile and firm in consistency. The rest of the examination was unremarkable. 

**Figure 1:**
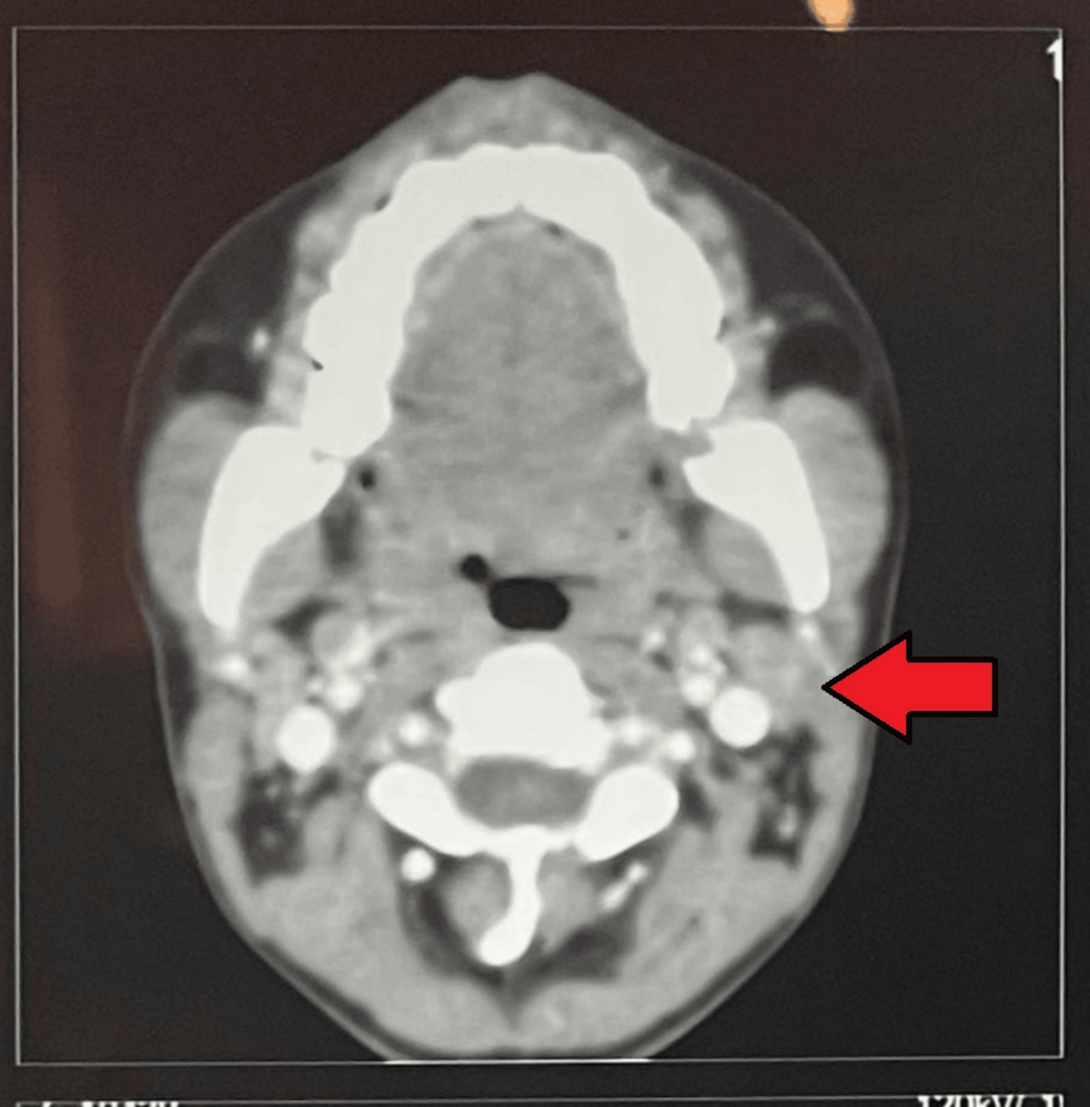
CT neck with contrast showing extensive lymphadenopathy, arrow points to enlarged lymph nodes, patient identifiers digitally concealed. CT: Computed axial tomographic scan.

**Figure 2:**
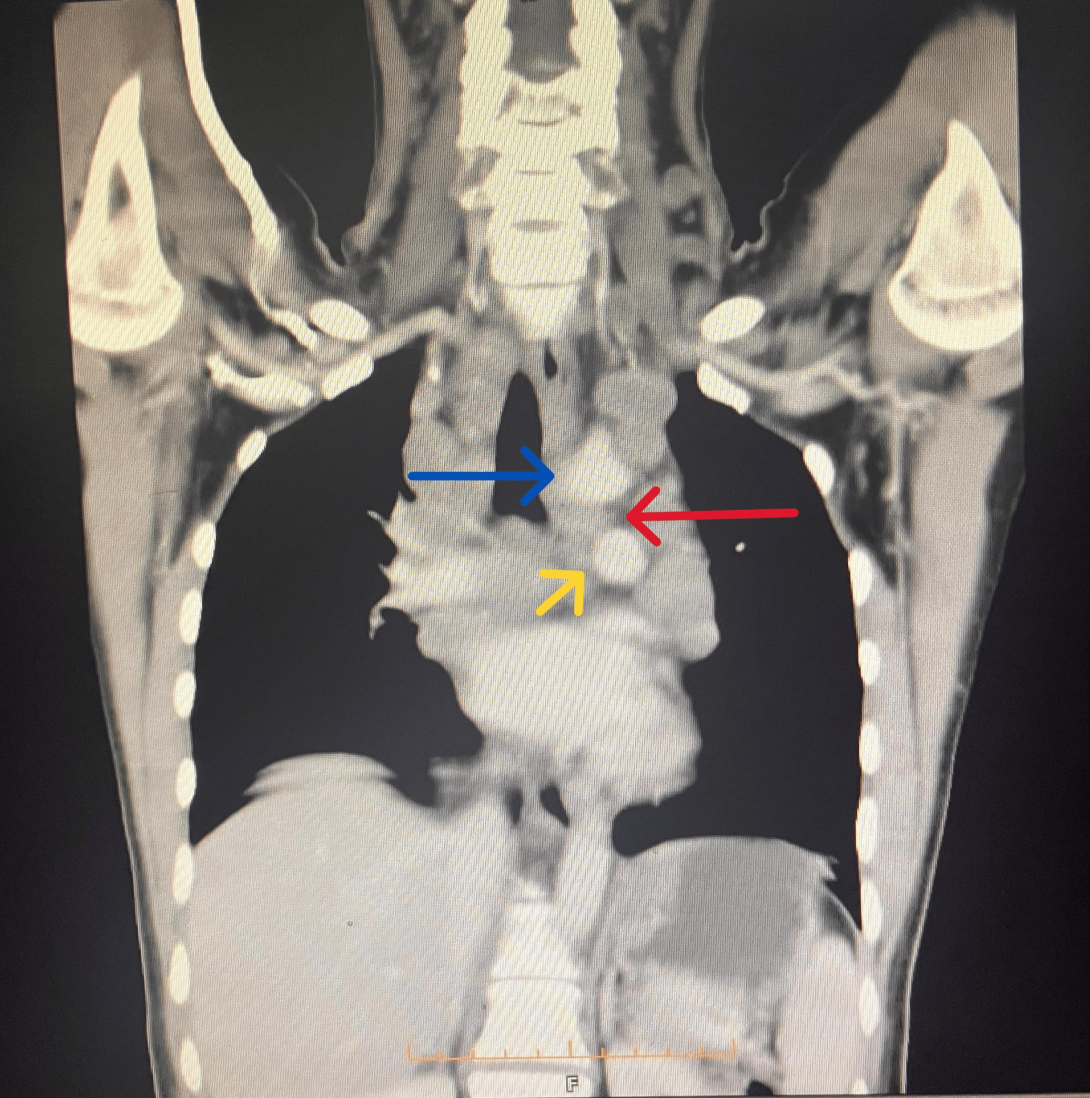
CT chest with contrast showing mediastinal lymphadenopathy. The blue arrow points to the aortic arch and the origin of the left subclavian artery, the yellow arrow points to the left pulmonary artery and the red arrow shows the lymph node present at the aortopulmonary window, likely compressing the left recurrent laryngeal nerve as it swings around the aortic arch. CT: Computerized tomographic scan.

Her complete blood count (CBC) showed hemoglobin levels of 9.0 gm/dl, mean corpuscular volume (MCV) 72 fL, mean corpuscular hemoglobin concentration (MCHC) 28 gm/dl, white blood cell count (WBC) 8300/µL, neutrophils 67%, lymphocytes 28%, and her erythrocyte sedimentation rate (ESR) was 115 mm in the first hour. All other baseline investigations, which included liver function tests, kidney function tests, serum electrolytes, urine complete exam and screening for hepatitis B and C, were within normal limits. Her chest x-ray revealed a widened mediastinum, which raised the suspicion of a mediastinal mass. A contrast-enhanced CT of the neck and chest was performed. CT neck showed multiple, bilateral, enlarged, conglomerate lymph nodes at levels IB, IIA, IIB, IVB, VA, and VB involving the anterior and posterior cervical regions. The largest confluent, conglomerate necrotic lymph node, measuring 3.0×2.2 cm, was identified in the right cervical region IVA. A few necrotic lymph nodes were noted in the left cervical region IVB (Figure 1). CT chest showed multiple enlarged lymph nodes in the mediastinum involving the paratracheal, pretracheal, perivascular, precarinal, and subcarinal regions. An enlarged lymph node was noticed at the aortopulmonary window, likely compressing the left recurrent laryngeal nerve. A few lymph nodes showed evidence of central necrosis. Lung parenchyma was normal bilaterally (Figure 2). A 70-degree rigid endoscopy was performed, which confirmed the presence of left vocal cord paralysis (Video 1). A left cervical lymph node excision biopsy was performed under local anesthesia. Histopathology revealed the presence of necrotizing granulomatous inflammation, with Langhan-type giant cells and horseshoe-shaped nuclei. Acid-fast bacilli (AFB) smear was negative. Following the histopathology reports, the patient was put on weight-based, first-line ATT. The patient weighed 38 kilograms, and she was initiated on a fixed-dose combination containing isoniazid 75 mg, rifampicin 150 mg, pyrazinamide 400 mg, and ethambutol 275 mg for two months. This was followed by isoniazid and rifampicin for the subsequent four months. When her weight increased, the dose was escalated accordingly. Culture and drug susceptibility testing were also performed on the lymph node excision biopsy specimen. The results became available after eight weeks and confirmed the presence of *Mycobacterium tuberculosis,* which showed sensitivity to all first-line anti-tuberculous drugs. As these findings were consistent with the initial histopathology, the patient was already on appropriate treatment, and ATT was continued accordingly. She was followed in the outpatient clinic every month.

**Video 1:** 70-degree rigid endoscopy showing left vocal cord palsy.

The patient tolerated the treatment well and had no adverse effects from ATT. Six months after the initiation of treatment, she gained four kilograms, her BMI improved from 16.4 kg/m² to 18.6 kg/m², and she reported a subjective improvement in her voice, which was also objectively appreciable. Her ESR improved to 25 mm in the first hour and her hemoglobin level was 11.5 gm/dl. Her cervical lymph nodes were impalpable after six months of treatment. Repeat CT neck and chest were performed, which showed a marked reduction in the overall size of the cervical nodes and some reduction in the size of the mediastinal nodes. The decision to continue ATT for another three months, with repeat *Mycobacterial *culture, and drug sensitivity testing was made. The decision to extend ATT was made based on clinical discretion, as a few mediastinal lymph nodes still showed evidence of necrosis and due to the slow clinical response. The patient's culture reports, along with extended drug sensitivity testing results, are awaited.

## Discussion

This case highlights an atypical presentation of tuberculosis. According to the WHO, approximately 510,000 new tuberculosis cases are reported annually in Pakistan [9]. Extrapulmonary tuberculosis accounts for 20 percent of all these cases [10]. While tuberculous lymphadenitis is common, hoarseness of voice being its sole presentation is rare [11,12]. Diagnosing such a case is difficult due to the absence of systemic symptoms and signs. Important differentials to be kept in mind should be malignancy and sarcoidosis [7,13].

Enlarged lymph nodes in tuberculosis can directly compress the recurrent laryngeal nerve, leading to hoarseness of voice [11]. This mostly occurs in cases of left-sided vocal cord palsy since the left recurrent laryngeal nerve has a longer course. As it swings around the aortic arch, there are higher chances of it getting compressed by enlarged nodes at the aortopulmonary window [8]. Hoarseness of voice could also occur due to the spread of caseous necrosis to the nerve by adjacent lymph nodes [14]. Cases of right-sided vocal cord palsy are caused mostly due to entanglement of the right recurrent laryngeal nerve by right lung apical fibrosis, secondary to pulmonary tuberculosis [14,15]. 

Our case was similar to the one reported from New Delhi, India, where a young patient presented with hoarseness of voice due to vocal cord paralysis and recurrent laryngeal nerve compression by lymph nodes at the aortopulmonary window [6]. Only a handful of cases with hoarseness of voice due to tuberculous mediastinal lymphadenopathy have been reported worldwide [8]. Out of them, very few had hoarseness of voice as the sole presenting feature [6]. We excluded the differentials of malignancy and sarcoidosis based on the histopathology, which showed the classic findings associated with *M. tuberculosis. *Our diagnosis was also supported by the presence of necrotic lymph nodes on CT imaging.* *The diagnosis was confirmed when the cultures came back positive, but the treatment had already been started by the time the cultures returned*.* After six months of therapy, the patient experienced improved appetite, weight gain, and partial improvement in her voice. The treatment outcome in our patient was similar to that reported by Vinatha et al. [12] and Daba and Canillas [16]. They too reported an improvement in clinical and radiological features accompanied by a partial improvement in the hoarseness of voice and vocal cord paralysis [12,16]. However, complete or near-complete recovery of voice has also been reported by several authors [6,11,14,17]. We decided to extend the treatment to a total of nine months. As per the National Tuberculosis Elimination Program (NTEP) guidelines, treatment duration in extrapulmonary tuberculosis can be extended beyond the standard six months on a case-by-case basis if there is a slow response [18].

Whether or not the hoarseness of voice might reverse depends on the mechanism of recurrent laryngeal nerve injury. If the nerve is merely compressed by the enlarged mediastinal nodes, treatment with ATT should result in regression in the size of the nodes and reversal of the nerve impingement. However, if the caseous necrosis has spread from the node to the adjacent nerve, or if the nerve has become entrapped in the apical fibrous pleura of the right lung, even the administration of ATT would not reverse the fibrous damage to the recurrent laryngeal nerve, and the hoarseness of voice and vocal cord paralysis would persist [11,17].

## Conclusions

This case highlights the importance of keeping in mind uncommon presentations of common diseases, like extrapulmonary tuberculosis presenting with the sole feature of hoarseness of voice. This is particularly true for areas with high disease endemicity. The case also highlights the importance of distinguishing lymph node tuberculosis from other diseases like lymphoma and sarcoidosis, which may present with similar symptoms and radiological signs. Histopathological and microbiological evidence should therefore be obtained whenever possible. Timely diagnosis and treatment of tuberculosis are essential to prevent morbidity and mortality.
